# The Effects of Interspersed Retrieval Practice in Multiple-List Learning on Initially Studied Material

**DOI:** 10.3389/fpsyg.2022.889622

**Published:** 2022-05-06

**Authors:** Oliver Kliegl, Verena M. Kriechbaum, Karl-Heinz T. Bäuml

**Affiliations:** Department of Experimental Psychology, University of Regensburg, Regensburg, Germany

**Keywords:** multiple-list task, testing effect, retrieval practice, initial study list, study material

## Abstract

The forward testing effect (FTE) refers to the finding that retrieval practice of previously studied material can facilitate retention of newly studied material more than does restudy of the material. The goal of the present study was to examine how such retrieval practice affects initially studied, unpracticed material. To this end, we used two commonly applied versions of the FTE task, consisting of either three (Experiment 1) or five (Experiment 2) study lists. While study of list 1 was always followed by an unrelated distractor activity, study of list 2 (3-list version) or lists 2, 3, and 4 (5-list version) was followed by either interim restudy or retrieval practice of the immediately preceding list. After studying all lists, participants were either asked to recall the first or last study list. Results showed that, for both the three-list and five-list versions, interim retrieval practice led to a typical FTE, irrespective of whether unrelated or categorized study lists were used. Going beyond the prior work, interim retrieval practice was found to have no effect on initially studied, unpracticed material, regardless of the type of study material. The findings suggest that using interim retrieval practice as a study method can improve recall of the last studied list without incurring a cost for the initially studied material. Our results are difficult to align with the view that retrieval practice induces context change, but are consistent with the idea that retrieval practice can lead participants to employ superior encoding strategies.

## Introduction

Retrieval practice of previously studied material can have tremendous benefits for the retention of the practiced material. A myriad of studies within the last 20 years have been able to demonstrate that repetition of studied material via active retrieval attempts often leads to dramatically better long-term memory of the material than restudy of the material does (e.g., Wheeler et al., 2003; Roediger and Karpicke, 2006). A growing number of both laboratory and field research supports the view that this testing effect, which has also been termed the backward testing effect, is a robust and general phenomenon that occurs across a wide range of study materials, age groups, and ability levels (for reviews, see Roediger and Butler, 2011; Karpicke, 2017).

A related line of research has demonstrated that retrieval practice not only benefits memory of the practiced material, but also promotes retention of the material studied later. In their landmark study, Szpunar et al. (2008) employed a multiple-list task in which participants studied five lists of words successively and were asked, immediately after study of lists 1–4, to solve either simple arithmetic tasks (distractor condition), study the word lists again (restudy condition), or attempt to retrieve the words from the immediately preceding list (retrieval-practice condition). Following study of list 5, participants were asked to recall as many list-5 words as possible. Relative to interim distractor activities or interim restudy, interim retrieval practice not only was found to increase the number of correctly recalled list-5 words but also to reduce the number of intrusions from lists 1 to 4 that were produced during this test. This beneficial effect of interspersed retrieval practice on final-list performance has been termed the forward testing effect (FTE) and has been found in both laboratory studies and educational settings. The generalizability of the FTE has been demonstrated for numerous types of study material, such as word lists, paired associates (Weinstein et al., 2011), prose material (Wissman et al., 2011), and videos (Szpunar et al., 2013). The FTE also has been observed across a variety of participant groups, including college students, children (Aslan and Bäuml, 2016), older adults (Pastötter and Bäuml, 2019), and individuals suffering from traumatic brain injury (Pastötter et al., 2013; for reviews, see Pastötter and Bäuml, 2014; Yang et al., 2018).

To date, it is unclear exactly which mechanism(s) mediate(s) the FTE. One promising explanation, however, is the context-change account (for an overview of further accounts, see Chan et al., 2018b; for multi-factor accounts, see also General Discussion below). This account assumes that during study of to-be-learned material, contextual features are encoded that are present when the material is acquired (Estes, 1955; Mensink and Raaijmakers, 1988). The critical idea is that retrieval activities interspersed between the study of single lists promote mental context change, and thus isolate newly from previously learned material (Shiffrin, 1970; Jang and Huber, 2008). At the time of test, this list isolation should reduce proactive interference from the earlier lists when the last studied list is recalled, thus enabling a more focused memory search for that list (Szpunar et al., 2008; Pastötter et al., 2011; Bäuml and Kliegl, 2013). Support for the context-change account, for instance, stems from studies showing that the FTE is characterized by shorter response latencies when a free-recall test of the critical final list is conducted (Bäuml and Kliegl, 2013; Lehman et al., 2014). Because response latencies in free-recall tests have been shown to indicate a smaller size of the mental search set (Wixted and Rohrer, 1993; Rohrer, 1996), the observation aligns with the context-change account's suggestion that interim retrieval practice promotes the isolation of the prior study lists from the critical last list.

## What are the effects of interim retrieval practice on the initially studied material?

FTE studies have thus far focused on the effects of retrieval practice on subsequently studied material. However, on both theoretical and practical grounds, it may be important to also assess how interim retrieval practice affects retention of the material studied earlier, such as the first studied list. From a theoretical perspective, examining the effects of interim retrieval practice on initially studied material, for instance, provides a critical test of the context-change account of the FTE. According to this account, list 1 retention primarily should be driven by the discrepancy in the study and text contexts which interim retrieval practice induces, and less by the improved discriminabilty between list 1 and later lists (e.g., Jang and Huber, 2008; Divis and Benjamin, 2014). Indeed, because the account assumes that interim retrieval practice increases context change across lists, there should be a greater mismatch between the list 1 study context and the later test context than in the absence of interim retrieval practice, thus leading to impaired list 1 recall at test. From a practical standpoint, it would be important to know if there was a net benefit to the learner in using interim retrieval practice as a study method, i.e., whether interim retrieval practice is still beneficial to memory when both the last list and the first list is taken into account. Indeed, if interim retrieval practice promoted the acquisition of the last studied list but caused forgetting of the first studied list, the suitability of interim retrieval practice as a valid study method might be questioned.

To date, only a single study has examined how interspersed retrieval activities affect the initially studied material (Divis and Benjamin, 2014). Divis and Benjamin applied a typical FTE task consisting of five lists with unrelated items, but with two critical modifications. First, instead of the standard (episodic) retrieval-practice task, participants engaged in an interim semantic-generation task in which they were either asked, between study of lists 1–4, to generate as many items as possible from a given semantic category that was unrelated to any items in the five study lists (e.g., SPORTS or PROFESSIONS). Second, after study of all five lists, half of the participants were asked to recall as many items as possible from the last studied list (list 5) while the remaining half of participants were asked to recall the initially studied list (list 1). Results showed that, like interim retrieval practice, interim semantic generation can enhance memory of the last studied list (see also, Pastötter et al., 2011), but can impair memory of the first-studied list. A subsequent experiment in which the researchers employed complex texts instead of word lists yielded a similar pattern, thus suggesting that the observed forgetting of the initially studied material may arise irrespective of study material.

The findings by Divis and Benjamin (2014) are consistent with the context-change account's assumption that interim retrieval activities promote contextual isolation of the study lists, thus making recall of the intially studied material harder. The observed list-1 forgetting also casts doubt on whether the combined effects of interim retrieval practice on prior and subsequent material lead to a net benefit, as the observed list-1 forgetting largely offset the list-5 enhancement effect. In addition, memory of lists 2–4—which the researchers did not asses—may also have suffered from retrieval-induced context change, making it possible that the list-5 enhancement effect is counterbalanced by forgetting effects of lists 1–4. However, Divis and Benjamin deviated from the typical FTE task because they used a semantic-generation task instead of an (episodic) retrieval-practice task, and it is unclear whether findings observed with this semantic version of the FTE task generalize to the episodic version of the FTE task (see Kliegl and Bäuml, 2021).

## The present study

The aim of the present study was to determine how retrieval practice conducted between the study of single item lists affects not only retention of the last but also retention of the first studied list. The results of four experiments are reported. Experiments 1a and 1b applied a variant of the three-list version of the FTE task (e.g., Pastötter et al., 2018; Kliegl and Bäuml, 2021) and Experiments 2a and 2b a variant of the five-list version of the task (e.g., Szpunar et al., 2008; Pastötter et al., 2011). The focus of the four experiments was on the questions of i) whether interim retrieval practice affects not only recall of the last studied item list but also recall of the initially studied item list, and ii) whether the results of Divis and Benjamin (2014) generalize from interim semantic generation to interim retrieval practice. In both experiments, the effects of interim retrieval practice were compared to the effects of interim restudy.

In their original study, Divis and Benjamin (2014) applied interim semantic generation rather than interim retrieval practice because semantic generation does not involve any practice of previously studied material. In fact, research on the backward testing effect (e.g., Roediger and Karpicke, 2006) suggests that retrieval practice on the first list enhances retention of the material by retrieving it. To avoid the potential confound of enhancing memory for list 1 in the present study, a different method was applied. In all four experiments, there was no retrieval practice (and no restudy) on list 1. Accordingly, in the three-list task of Experiments 1a and 1b, there was practice on list 2 only, and in the five-list task of Experiments 2a and 2b, there was practice on lists 2–4. This procedure allows evaluation of the effects of interim retrieval practice on both the first and the last studied lists without enhancing recall of the materials by retrieving them.

## Experiments 1a and 1b

Experiments 1a and 1b sought to examine how interim retrieval practice affects memory of initially studied information in a three-list version of the FTE task. In this task, participants were always asked to solve simple arithmetic problems after study of list 1 and, after study of list 2, either immediately restudied list 2 (restudy condition) or retrieved as many list-2 items as possible in a free-recall test (retrieval-practice condition). Following study of list 3, all participants engaged in a free-recall test of either the initially studied list 1 or the last studied list 3. For this test, both recall totals and intrusions were measured. In Experiment 1a, participants studied unrelated item lists, while in Experiment 1b, they studied categorized item lists. Prior research shows that both types of study material are well suited to ensure the buildup of proactive interference across study lists (Szpunar et al., 2008). Furthermore, recent work indicates that interim retrieval practice can have different effects on subsequently studied material for unrelated and categorized material (Kliegl and Bäuml, 2021) ^1^ and, therefore, the present study examined whether or not study material can also influence the effects of interim retrieval practice on *initially* studied lists.

The results of Experiments 1 and 1b will show whether the effects reported by Divis and Benjamin (2014) still transpire when interim retrieval practice is applied in lieu of interim semantic generation. If so, interim retrieval practice should not only facilitate memory of the last studied list but also impair retention of the first studied list. In addition, if the effects of interim retrieval practice on the initial material also generalized across different types of study material, then list-1 forgetting should arise both when unrelated (Experiment 1a) and when categorized (Experiment 1b) study lists are employed.

###  Method

#### Participants

The required sample size in Experiments 1 and 2 was calculated using G^*^Power (Version 3.1.9.2; Faul et al., 2009). In particular, based on the meta-analytic effect size estimate for the FTE in two-groups designs (Hedges' *g* = 0.84, Chan et al., 2018b), *n* = 26 are required for each between-subject condition to achieve a power of 1-β = 0.80 for the two-groups F test, given α = 0.05.

Closely following this recommendation, we recruited 120 students at Regensburg University for Experiment 1a (mean age = 24.7 years) and 120 students at Regensburg University for Experiment 1b (mean age = 23.6 years), with 30 participants in each experiment's four experimental conditions. Participants took part in the experiments in return for either partial course credit or a compensatory amount of money. All participants spoke German as their native language. All reported experiments were carried out in accordance with the provisions of the World Medical Association Declaration of Helsinki. Participants in both experiments were tested individually and online via Zoom.

#### Material

For Experiment 1a, a set of 72 unrelated German nouns of medium frequency was drawn from the CELEX database (Duyck et al., 2004). ^2^ For each participant, items were assigned randomly to three lists consisting of 24 items each. The study material was identical to the material applied in one of our earlier studies (Kliegl and Bäuml, 2021, Experiments 1a, 2a, 3a). For Experiment 1b, a set of 72 German nouns was drawn from the Van Overschelde et al. (2004) category norms, which consisted of 12 exemplars from six categories. The six categories were BUILDING PARTS, KITCHEN UTENSILS, BODY PARTS, MUSICAL INSTRUMENTS, WEATHER PHENOMENONS, and TYPES OF FABRIC. Items' average taxonomic frequencies did not differ between categories, *F*_(5, 66)_ < 1. For each participant, four exemplars of each of the six categories were assigned randomly to three lists, resulting in 24 items per list.

Again, the study material was identical to the material applied in one of our earlier studies (Kliegl and Bäuml, 2021, Experiments 1b, 2b, 3b).

#### Design

Both experiments had a 2 x 2 between-participants design with the factors PRACTICE TYPE (restudy, retrieval practice), and CRITICAL LIST (first list, last list). After study of list 2, participants were either immediately asked to restudy list 2 (restudy condition) or to recall as many list-2 words as possible (retrieval-practice condition). Finally, conditions differed in whether, following study of list 3, participants were asked to recall as many words as possible of list 1 (first-list condition) or list 3 (last-list condition).

#### Procedure

Prior to the start of both experiments, participants were told that they would be asked to study several lists of items. They were also informed that they should anticipate various activities that may follow the presentation of each single list, which can include simple arithmetic tasks, restudy of a list that they had just previously studied, or a free-recall test on all the words from a just studied list. It was pretended that these interlist activities would occur on a completely random basis when, in fact, interlist activities differed between conditions, with participants in the restudy group restudying list 2 after studying that list for the first time, and participants in the retrieval-practice group recalling list 2 after studying that list. Participants were also made aware that, regardless of these interlist activities, all study lists would be tested in a final cumulative test.

At the start of Experiments 1a and 1b, the items of the three lists were visually presented at the center of a computer screen, and the 24 words of each list were exposed individually for 4.5 s with a 0.5 s interitem interval. After the presentation of each single list, participants counted backward in steps of threes from a random three-digit number for 30 s. Experimental conditions differed in the type of interlist activity that followed this backward counting after lists 1 and 2. participants were always asked to solve simple arithmetic tasks for 2 min following study of list 1 but, following study of list 2, they either were asked to study list 2 once again (restudy condition) or were given 120 s to recall as many list-2 items as possible(retrieval-practice condition). After study of list 3 and the backward-counting task, participants in the first-list condition were asked to say out loud as many items as possible of list 1, while participants in the last-list condition were asked to say out loud as many items as possible of list 3. They were given 120 s for this free-recall task. Following recall of the critical list and 5 min of playing Tetris, participants had 6 min to write down as many words as possible from all three study lists in a final cumulative test. No feedback was provided during or after any of the free-recall tests. Final-test performance of lists 1 to 3 is of no direct relevance for the present study and will not be reported.

## Results of Experiment 1a

For all experiments, we provide Bayes factors (B_0__1_)—which reflect the odds in favor of the null hypothesis over the alternative hypotheses—when a finding did not reach conventional level of statistical significance (i.e., α = 0.05). For general orientation, a B_0__1_ ranging from 1 to 3 can be considered as anecdotal evidence for the null hypothesis, a B_0__1_ ranging from 3 to 10 as moderate evidence for the null hypothesis, and a B_0__1_ ranging from 10 to 30 as strong evidence for the null hypothesis (Raftery, 1995; Masson, 2011).

###  Correct Recall

**Figure 1:**
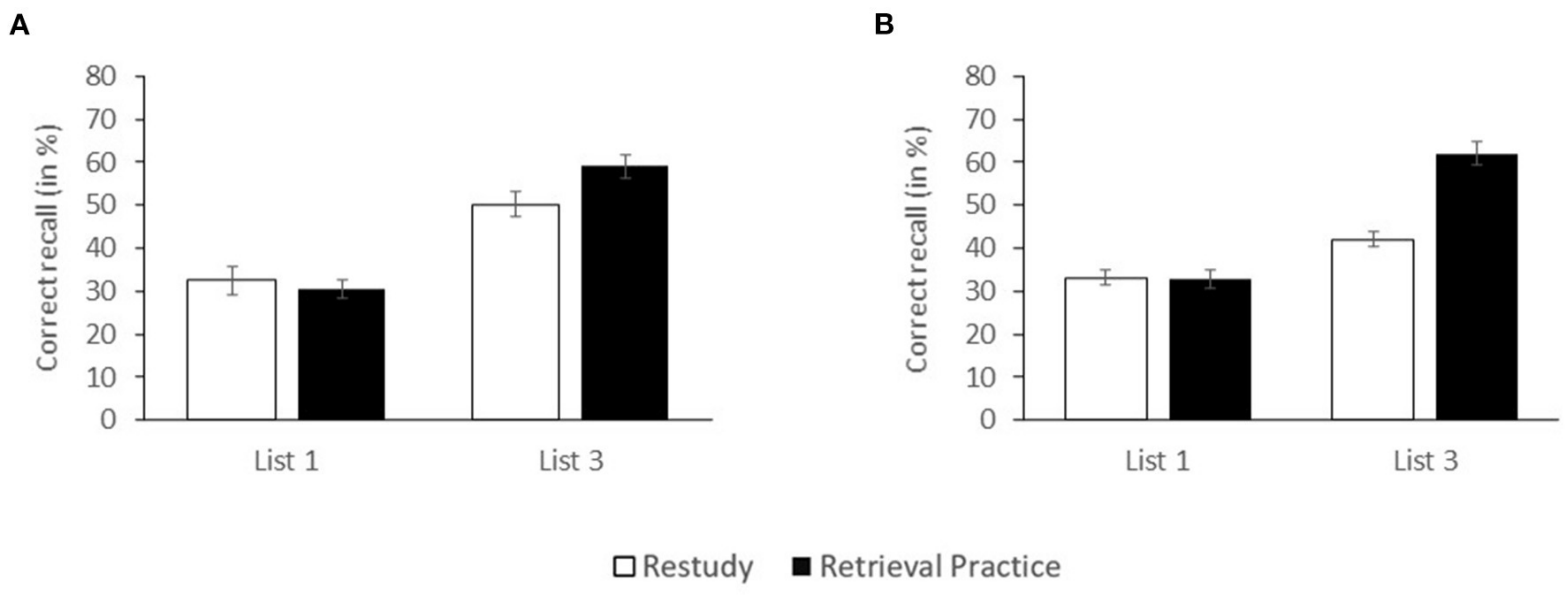
Recall rates of first-list and last-list (list 3) items as a function of PRACTICE for **(A)** unrelated study lists (Experiment 1a) and **(B)** categorized study lists (Experiment 1b). Error bars reflect standard errors.

In Figure 1A, the percentage of correctly recalled critical items is shown as a function of PRACTICE (restudy, retrieval practice) and CRITICAL LIST (first list, last list). A 2 x 2 ANOVA with the two factors revealed no main effect of PRACTICE, *F*_(1, 116)_=1.529, *MSE* = 0.022, *p* = 0.219, partial η^2^ = 0.013, B_0__1_ = 5.031, but a significant main effect of CRITICAL LIST, *F*_(1, 116)_=71.795, *MSE* = 0.022, *p* < 0.001, partial η^2^ = 0.382, reflecting that overall, recall was higher for last-list than first-list items (54.8 vs. 31.6%). There was also a significant interaction between factors, *F*_(1, 116)_=4.115, *MSE* = 0.022, *p*=0.045, partial η^2^ = 0.034, reflecting that the interim practice format affected first-list items differently than last-list items. Indeed, while for last-list items, pairwise comparisons showed superior recall performance following retrieval practice relative to restudy (59.2 vs. 50.3%), *F*_(1, 58)_=5.379, *MSE* = 0.022, *p* = 0.024, Cohen's *d* = 0.596, thus reflecting the typical FTE, for first-list items, pairwise comparisons showed no difference between the two practice conditions (30.5 vs. 32.7%), *F*_(1, 58)_ < 1, Cohen's *d* = 0.145, B_0__1_ = 6.605.

#### Intrusions

In Experiments 1a and 1b, all items from the two non-critical lists that participants produced during the recall test of the critical list were counted as intrusions. A 2 x 2 ANOVA with the two factors of PRACTICE and CRITICAL LIST revealed no main effects of PRACTICE, *F*_(1, 116)_ < 1, B_0__1_ = 8.042, or CRITICAL LIST, *F*_(1, 116)_ < 1, B_0__1_ = 10.140, and no interaction between factors, *F*_(1, 116)_ = 2.398, *MSE* = 0.890, *p* = 0.124, partial η^2^ = 0.020, B_0__1_ = 3.210 (see Table 1).

**Table 1:** Mean number of list-3 intrusions for Experiments 1 and 2 (standard errors are shown in parenthesis).

**Critical list**	**First list**		**Last list**	
**Condition**	**Restudy**	**Retrieval practice**	**Restudy**	**Retrieval practice**
Experiment 1a	0.47 (0.14)	0.60 (0.22)	0.67 (0.20)	0.27 (0.10)
Experiment 1b	1.60 (0.26)	1.43 (0.27)	1.23 (0.31)	0.70 (0.15)
Experiment 2a	0.93 (0.29)	0.77 (0.27)	0.57 (0.17)	0.20 (0.07)
Experiment 2b	1.50 (0.29)	1.10 (0.27)	1.07 (0.18)	0.57 (0.16)

## Results of Experiment 1b

###  Correct Recall

In Figure 1B, the percentage of correctly recalled critical items is shown as a function of PRACTICE (restudy, retrieval practice) and CRITICAL LIST (first list, last list). A 2 x 2 ANOVA with the two factors revealed main effects of PRACTICE, *F*_(1, 116)_=21.291, *MSE* = 0.014, *p* < 0.001, partial η^2^ = 0.155, and CRITICAL LIST, *F*_(1, 116)_=80.889, *MSE* = 0.014, *p* < 0.001, partial η^2^ = 0.411, reflecting that overall, recall was higher following retrieval practice than restudy (47.5 vs. 37.7%) and for last-list than first-list items (52.2 vs. 33.0%). There was also a significant interaction between factors, *F*_(1, 116)_=22.759, *MSE* = 0.014, *p* < 0.001, partial η^2^ = 0.164, reflecting that the interim practice format affected first-list items differently than last-list items. Indeed, while for last-list items, pairwise comparisons showed superior recall performance following retrieval practice relative to restudy (62.2 vs. 42.2%), *F*_(1, 58)_=36.046, *MSE* = 0.017, *p* < 0.001, Cohen's *d* = 1.713, thus reflecting the typical FTE, for first-list items, pairwise comparisons showed no difference between the two practice conditions (32.8 vs. 33.1%), *F*_(1, 58)_ < 1, Cohen's *d* = 0.029, B_0__1_ = 7.746.

#### Intrusions

A 2 x 2 ANOVA with the two factors of PRACTICE and CRITICAL LIST revealed only a main effect of CRITICAL LIST, *F*_(1, 116)_=4.695, *MSE* = 1.933, *p*=0.032, partial η^2^ = 0.039, reflecting that, overall, number of intrusions was higher for first-list than last-list items (1.52 vs. 0.97). There was, however, no main effect of PRACTICE, *F*_(1, 116)_=1.901, *MSE* = 1.933, *p*=0.171, partial η^2^ = 0.016, B_0__1_ = 4.130, and no interaction between factors, *F*_(1, 116)_ < 1, B_0__1_ = 8.370 (see Table 1).

## Additional Analysis

The results of Experiments 1a and 1b suggest that, for both unrelated and categorized study lists, interim retrieval practice induces not only an FTE but also leaves memory of initially studied material unaffected. We examined more directly whether study material had any impact on the effects of interim retrieval practice on subsequently and initially studied material. To this end, we pooled the data of Experiments 1a and 1b to conduct a 2 x 2 x 2 ANOVA with the three factors of STUDY MATERIAL, PRACTICE and CRITICAL LIST. ANOVA revealed no interaction between the three factors, *F*_(1, 232)_=1.772, *MSE* = 0.018, *p*=0.184, partial η^2^ = 0.008, B_0__1_ = 6.212, no two-way interactions between the factors of STUDY MATERIAL and CRITICAL LIST, *F*_(1, 232)_=1.341, *MSE* = 0.018, *p* = 0.248, partial η^2^ = 0.006, B_0__1_ = 7.801, and the factors of STUDY MATERIAL and PRACTICE, *F*_(1, 232)_=3.459, *MSE* = 0.018, *p* = 0.064, partial η^2^ = 0.015, B_0__1_ = 2.654, and no main effect of MATERIAL, *F*_(1, 232)_ < 1, B_0__1_ = 14.629. The results of the three-way ANOVA thus suggest that the effects of interim retrieval practice on subsequently and initially studied material were largely independent of study material^3^

###  Discussion

The results of Experiment 1a and 1b replicate prior work by demonstrating that interim retrieval practice can induce an FTE both when unrelated (Experiment 1a) and when categorized (Experiment 1b) study lists are used (Szpunar et al., 2008; Kliegl and Bäuml, 2021). More important, neither Experiment 1a nor Experiment 1b found any effects of interim retrieval practice on the initially studied list-1 items. These findings contrast with the Divis and Benjamin (2014) study, which showed that when the interim retrieval activity consists of a semantic-generation task, reliable forgetting of list-1 items can arise, suggesting that interim retrieval practice may differ in its effects on initially studied material from interim semantic generation.

Results showed no reliable effects of practice format on number of first-list and last-list intrusions. This may seem surprising since interim retrieval practice often results in reduced number of intrusions for the last-list items (e.g., Szpunar et al., 2008; Chan et al., 2018a). Table 1 suggests that, at least numerically, fewer intrusions were made following retrieval practice than restudy in both Experiment 1a (0.27 vs. 0.67) and 1b (0.70 vs. 1.23). However, since the number of intrusions was already relatively low in the restudy condition, there was not much room for a further reduction in number of intrusions (see also Kliegl and Bäuml, 2021, for similar observations).

## Experiments 2a and 2b

The goal of Experiments 2a and 2b was to examine whether the findings of Experiments 1a and 1b generalize from the three-list to the five-list FTE task. The number of study lists could be critical for results because the three-list task that was used in Experiments 1a and 1b only involved a single retrieval-practice or restudy period (after study of list 2), whereas in the five-list task used in Experiments 2a and 2b, participants engaged in a total of three retrieval-practice or restudy periods (i.e., after study of list 2, list 3, and list 4). Following the context-change account of the FTE, an increase in interim retrieval activities should render the context present when list 1 is tested more distinct from the context present at encoding of list 1, thus making it more difficult to recall list-1 items at test. The absence of list-1 forgetting following interim retrieval practice as observed in Experiments 1a and 1b thus might be due to the single retrieval-practice period employed, and forgetting of list-1 items might arise in Experiments 2a and 2b when retrieval practice is extended to further lists.

###  Methods

#### Participants

On the basis of the estimate reported in Experiment 1, we recruited 120 students at Regensburg University for both Experiments 2a and 2b (mean age = 23.6 years in both experiments).

#### Material, Design, and Procedure

Experimental details of Experiments 2a and 2b were similar to Experiments 1a and 1b, with the major exception that five study lists consisting of 15 items per list were used instead of three study lists consisting of 24 items per list. As a result, in Experiment 2a, we added three additional German nouns of medium frequency drawn from the CELEX database (Duyck et al., 2004) to the 72 items used in Experiment 1 to get to a total of 75 items. In Experiment 2b, each categorized list now consisted of three exemplars from five out of the six categories used in Experiment 1b (the category KITCHEN UTENSIL was omitted). To obtain a total number of 15 exemplars per category, we added three exemplars from the Van Overschelde et al. (2004) norm to the 12 existing exemplars used in Experiment 1b. Another consequence of applying five, instead of three lists, was that in both Experiments 2a and 2b, interim restudy and interim retrieval practice of the preceding list was now conducted following study of lists 2, 3, and 4.

## Results

###  Experiment 2a

#### Correct Recall

**Figure 2:**
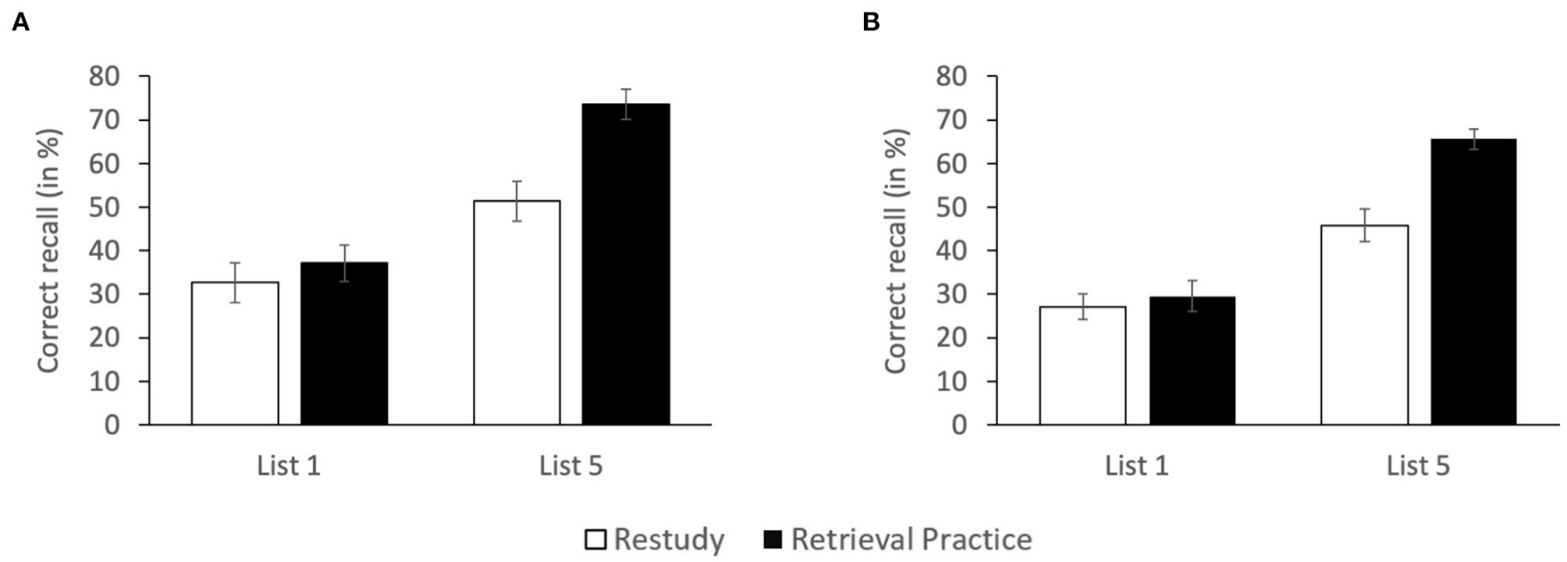
Recall rates of first-list and last-list (list 5) items as a function of PRACTICE for **(A)** unrelated study lists (Experiment 2a) and **(B)** categorized study lists (Experiment 2b) Error bars reflect standard errors.

In Figure 2A, the percentage of correctly recalled critical items is shown as a function of PRACTICE (restudy, retrieval practice) and CRITICAL LIST (first list, last list). A 2 x 2 ANOVA with the two factors revealed main effects of PRACTICE, *F*_(1, 116)_ = 9.905, *MSE* = 0.054, *p* = 0.002, partial η^2^ = 0.079, and CRITICAL LIST, *F*_(1, 116)_ = 42.306, *MSE* = 0.054, *p* < 0.001, partial η^2^ = 0.267, reflecting that, overall, recall was higher following retrieval practice than restudy (55.3 vs. 42.0%) and for last-list than first-list items (62.4 vs. 34.9%). There was also a significant interaction between factors, *F*_(1, 116)_=4.402, *MSE* = 0.054, *p* = 0.038, partial η^2^ = 0.037, reflecting that the interim practice format affected first-list items differently than last-list items. Indeed, while for last-list items, pairwise comparisons showed superior recall performance following retrieval practice relative to restudy (73.6 vs. 51.3%), *F*_(1, 58)_ = 14.707, *MSE* = 0.050, *p* < 0.001, Cohen's *d* = 0.958, thus reflecting the typical FTE, for first-list items, pairwise comparisons showed no difference between the two practice conditions (37.1 vs. 32.7%), *F*_(1, 58)_ < 1, Cohen's *d* = 0.192, B_0__1_ = 5.916.

#### Intrusions

In Experiments 2a and 2b, all items from the four non-critical lists that participants produced during the recall test of the critical list were counted as intrusions. A 2 x 2 ANOVA with the two factors of PRACTICE and CRITICAL LIST revealed only a main effect of CRITICAL LIST, *F*_(1, 116)_=4.527, *MSE* = 1.443, *p* = 0.035, partial η^2^ = 0.038, reflecting that, overall, number of intrusions was higher for first-list than last-list items (0.85 vs. 0.38). There was, however, no main effect of PRACTICE *F*_(1, 116)_=1.478, *MSE* = 1.443, *p* = 0.227, partial η^2^ = 0.013, B_0__1_ = 5.125, and no interaction between factors, *F*_(1, 116)_ < 1, B_0__1_ = 9.839 (see Table 1).

###  Experiment 2b

#### Correct Recall

In Figure 2B, the percentage of correctly recalled critical items is shown as a function of PRACTICE (restudy, retrieval practice) and CRITICAL LIST (first list, last list). A 2 x 2 ANOVA with the two factors revealed main effects of PRACTICE, *F*_(1, 116)_=12.040, *MSE* = 0.031, *p* = 0.001, partial η^2^ = 0.094, and CRITICAL LIST, *F*_(1, 116)_ = 72.862, *MSE* = 0.031, *p* < 0.001, partial η^2^ = 0.386, reflecting that, overall, recall was higher following retrieval practice than restudy (47.6 vs. 36.4%) and for last-list than first-list items (55.7 vs. 28.3%). There was also a significant interaction between factors, *F*_(1, 116)_ = 7.325, *MSE* = 0.031, *p* = 0.008, partial η^2^ = 0.059, reflecting that the interim practice format affected first-list items differently than last-list items. Indeed, while for last-list items, pairwise comparisons showed superior recall performance following retrieval practice relative to restudy (65.6 vs. 45.8%), *F*_(1, 58)_ = 19.652, *MSE* = 0.030, *p* < 0.001, Cohen's *d* = 1.128, thus reflecting the typical FTE, for first-list items, pairwise comparisons showed no difference between the two practice conditions (29.6 vs. 27.1%), *F*_(1, 58)_ < 1, Cohen's *d* = 0.139, B_0__1_ = 6.690.

#### Intrusions

A 2 x 2 ANOVA with the two factors of PRACTICE and CRITICAL LIST revealed only a main effect of CRITICAL LIST, *F*_(1, 116)_ = 4.337, *MSE* = 1.616, *p* = 0.039, partial η^2^ = 0.036, reflecting that, overall, number of intrusions was higher for first-list than last-list items (1.30 vs. 0.82). There was, however, no main effect of PRACTICE *F*_(1, 116)_ = 3.760, *MSE* = 1.616, *p* = 0.055, partial η^2^ = 0.031, B_0__1_ = 1.616, and no interaction between factors, *F*_(1, 116)_ < 1, B_0__1_ = 10.694 (see Table 1).

###  Additional Analysis

Analoguous to Experiments 1a and 1b, it was examined more directly whether study material had any impact on the effects of interim retrieval practice on subsequently and initially studied material by pooling the data of Experiments 2a and 2b. A 2 x 2 x 2 ANOVA with the factors of STUDY MATERIAL, PRACTICE, and CRITICAL LIST revealed no interaction between the three factors, *F*_(1, 232)_ < 1, B_0__1_ = 15.478, no two-way interactions between the factors of STUDY MATERIAL and CRITICAL LIST, *F*_(1, 232)_ < 1, B_0__1_ = 15.479, and the factors of STUDY MATERIAL and PRACTICE, *F*_(1, 232)_ < 1, B_0__1_ = 14.222. There was only a main effect of STUDY MATERIAL, *F*_(1, 232)_ = 6.304, *MSE* = 0.042, *p* = 0.013, partial η^2^ = 0.026, reflecting that overall recall performance was higher for unrelated than categorized material (48.7 vs. 42.0%). Analogous to Experiments 1a and 1b, the results of the three-way ANOVA suggest that the effects of interim retrieval practice on subsequently and initially studied material are independent of study material.

###  Discussion

The results of Experiments 2a and 2b generalize the findings of Experiments 1a and 1b, by showing that for both unrelated and categorized study lists, interim retrieval practice can induce an FTE but leave memory of list-1 material largely unaffected. The experiments thus provide another demonstration that the effects of interim retrieval practice may differ from the effects of interim semantic generation on initially studied material. Indeed, even though Experiment 2a followed Divis and Benjamin's (2014) Experiment 1 by using unrelated study material and a five-list task, results did not replicate the list-1 forgetting effect that Divis and Benjamin observed in response to interim semantic generation.

Similar to Experiments 1a and 1b, there was no reliable evidence that practice format affected the number of first-list and last-list intrusions. Regarding last-list items, Table 1 again suggests that fewer intrusions were produced following retrieval practice than restudy in both Experiments 2a (0.20 vs. 0.57) and 2b (0.57 vs. 1.07). Due to the relatively low number of intrusions in the restudy condition, not much room was left for any further reductions in the number of intrusions (see also Kliegl and Bäuml, 2021; for similar observations).

## General Discussion

In the two experiments presented here, no evidence was found that interim retrieval practice affected recall of initially studied, unpracticed material in multiple-list learning. While interim retrieval practice led to a typical FTE in both experiments, recall performance of list-1 items was highly similar in response to interim restudy practice and interim retrieval practice. This held regardless of i) whether the three-list version (Experiments 1a and 1b) or the five-list version (Experiments 2a and 2b) of the FTE task was employed, and ii) whether unrelated (Experiments 1a and 2a) or categorized (Experiments 1b and 2b) study lists were applied.

The present results are difficult to align with the context-change account of the FTE which predicts that interim retrieval activities should accelerate contextual drift and thus increase the dissimilarity between the context at encoding and the context at test, which should cause impaired recall of the first studied material. These findings are also inconsistent with another context-based explanation of the FTE, the postretrieval monitoring account (Hunt et al., 2011; Pierce et al., 2017). This account assumes that interim retrieval practice updates the internal context in which the retrieved information is embedded, so that the retrieved items are associated with both a study and a retrieval context, whereas studied items are associated with a study context only. Following this assumption, interim retrieval practice should lead to the FTE because interim retrieval practice should make the last studied (unretrieved) list easier to distinguish from the other (retrieved) lists on the basis of their context differences. Analoguously, interim retrieval practice should also enhance recall of an unretrieved first list from the subsequent (retrieved) lists. The present findings obviously do not align with this prediction, showing only enhanced recall of the last studied, but not first studied, list.

In contrast, the findings are consistent with a third explanation of the FTE, the strategy-change account. This explanation assumes that interim retrieval practice leads participants to consider new—and potentially more effective—strategies for further learning. Retrieval practice can indeed provide critical information about the learning task at hand, and may enable participants to build expectations about the particular format of later tests or the presence of retrieval cues on these tests. On the basis of such information, encoding strategies may be optimized (Soderstrom and Bjork, 2014; Davis and Chan, 2015; Chan et al., 2018a). Naturally, retrieval practice would only be able to enhance encoding strategies for material studied after retrieval practice, and thus, should leave list-1 retention unaffected, which fits the findings observed in the present four experiments. While the present work did not directly test the strategy-change explanation, several recent studies analyzing clustering scores have provided further evidence that interim retrieval practice can induce strategy change for both unrelated and categorized word lists. Studies analyzing temporal clustering scores for unrelated word lists have demonstrated that interim retrieval practice improves strategic processing of temporal order information (Yang et al., 2020, in press), and studies analyzing semantic clustering scores for categorized word lists have shown that interim retrieval practice results in a stronger propensity to cluster retrieval based on category membership (Chan et al., 2018a, 2020; Kliegl and Bäuml, 2021). ^4^

The current results suggest that Divis and Benjamin's (2014) finding that interim semantic generation can induce forgetting of initially studied material does not generalize to interim retrieval practice. The difference in effects between interim retrieval practice and interim semantic generation on list-1 recall, however, is not completely surprising. While both types of retrieval activities have been found to be able to induce an FTE (Pastötter et al., 2011; Divis and Benjamin, 2014), recent research has demonstrated that the two types of retrieval activities can have different effects on subsequently studied material (Kliegl and Bäuml, 2021). For instance, while both types of retrieval activities can induce an FTE when unrelated study lists are used, for categorized lists, only interim retrieval practice, but not interim semantic generation, led to an FTE. Thus, apparently, retrieval practice and semantic generation can differ both in their effects on the last studied material and in their effects on the first studied material. Retrieval practice and semantic generation thus cannot be regarded equivalent for recall in multiple-list learning.

The observations that Divis and Benjamin (2014) made by applying interim semantic generation suggest that retrieval activities can be accompanied by benefits *and* costs for the last and first studied list. In contrast, the present findings with interim retrieval practice do not provide any evidence of cost effects and show only a benefit for the last studied list. Critically, our experiments did not involve any practice of the first studied list to avoid that possible cost effects could be masked by enhancement effects resulting from retrieval practice of this list. The question therefore arises whether applying interim retrieval practice after study of list 1 would have altered the outcome. Previous research on the backward testing effect (e.g., Roediger and Karpicke, 2006) suggests that retrieval practice of list 1 could lead to a recall enhancement for list 1, and thus to improved memory of both the initially studied and last studied materials. However, given that the backward testing effect is often quite small or even absent when the retention interval between study and test is relatively short (e.g., Rowland, 2014), retrieval practice of list 1 may also leave recall unaffected. Future work may examine whether an opportunity to practice list 1 leads to a different pattern of results than was observed here.

From a practical perspective, the present findings thus suggest that applying interim retrieval practice as a study technique may yield a net-benefit, with recall enhancement for the last list and a neutral effect for the first list. Granted, the current study is only a first attempt to explore the extent to which interim retrieval practice affects recall and future work should also examine the effects of retrieval practice on (all) the intermediate lists. Such work may also apply complex texts as study material, thus coming up with a more elaborate picture of how interim retrieval practice affects memory of all previously studied materials.

*To conclude*, the present two experiments showed that, for both the three-list and five-list versions of the FTE task, interim retrieval practice did not induce any forgetting of initially studied material, regardless of whether unrelated or categorized study lists were used. Theoretically, the findings align with the strategy-change account of the FTE, but are difficult to align with the context-based accounts of the FTE. Empirically, the findings underscore the suitability of interim retrieval practice as an efficient study method which seems to promote memory of subsequently studied material without affecting retention of the initially studied material.

## Data Availability Statement

The datasets presented in this study can be found in online repositories. The names of the repository/repositories and accession number(s) can be found below: https://osf.io/em75n/.

## Ethics Statement

Ethical review and approval was not required for the study on human participants in accordance with the local legislation and institutional requirements. Written informed consent for participation was not required for this study in accordance with the national legislation and the institutional requirements.

## Author Contributions

K-HB and OK developed the study concept and experimental design. OK organized the data collection, performed the data analysis, and drafted the manuscript. VK and K-HB gave critical input for various revisions of the manuscript. All authors approved the final version of the manuscript for submission.

## Funding

This publication was funded by the Open Access Fund of Universität Regensburg and the German Research Foundation (DFG) within the Open Access Publishing funding program. This work was supported by a grant from the German Research Foundation (Deutsche Forschungsgemeinschaft, DFG) awarded to K-HB (BA 1382/18-1).

## Conflict of Interest

The authors declare that the research was conducted in the absence of any commercial or financial relationships that could be construed as a potential conflict of interest.

## Publisher's Note

All claims expressed in this article are solely those of the authors and do not necessarily represent those of their affiliated organizations, or those of the publisher, the editors and the reviewers. Any product that may be evaluated in this article, or claim that may be made by its manufacturer, is not guaranteed or endorsed by the publisher.

## Footnotes

^1^Kliegl and Bäuml (2021) found that, for instance, when the retention interval between study and test of the final list was prolonged or the lag prior to study of the final list was prolonged, an FTE only arose for unrelated, but not for categorized, study material.

^2^All item materials that were applied in the present experiments as well as all data are available via osf.io/k4y86/.

^3^The marginally significant interaction between STUDY MATERIAL and PRACTICE does suggest that study material had at least some relevance for the effects of practice format on critical-list recall. Indeed, when the same ANOVA was restricted to last-list items, a significant interaction arose between the two factors, F_(1, 116) = 4.723, MSE=0.019, p=0.032, *partial*η^2^ = 0.039, suggesting a smaller FTE for unrelated than categorized material (8.9 vs. 20.0%). When the ANOVA was restricted to first-list items, however, there was no interaction between the two factors, *F*_(1, 116)_ < 1, B_0__1_ = 9.979, suggesting that the differences between the restudy and retrieval-practice conditions were similar for unrelated and categorized material (0.3 vs. 2.2%).

^4^More recently, Chan et al. (2020) pointed out that interim retrieval practice may induce not only changes in encoding strategy, but also retrieval strategy. Extending the strategy-change account by including this assumption leads to less clear-cut expectations for how interim retrieval practice affects list-1 retention because improved retrieval strategies might also lead to enhanced list-1 recall, which differs from the present results.
